# Looking up to Look Within: Preliminary Findings of the Restorative Effects of Astronomical Retreats in South Africa

**DOI:** 10.3390/ijerph23081016

**Published:** 2026-08-04

**Authors:** Dominic Gregory Vertue, Nicole Thomas, Therese Fish, Charles Takalana, Kevin Govender, Sally Ann Macfarlane, Lynn Hendricks

**Affiliations:** 1 South African Astronomical Observatory (SAAO), Observatory, Cape Town 7925, South Africa; charles@astro4dev.org (C.T.); kg@astro4dev.org (K.G.); 2 Office of Astronomy for Development, International Astronomical Union, Observatory 7925, South Africa; 3 CoCREATE Research Equity Hub, Division of Health Systems and Public Health, Department of Global Health, Faculty of Medicine and Health Sciences, Stellenbosch University Tygerberg Campus, Parow 7505, South Africa; nthomas@sun.ac.za (N.T.); tfish@sun.ac.za (T.F.); lynnah@sun.ac.za (L.H.); 4 Department of Physics, Stellenbosch University, Matieland 7602, South Africa; 5 Inter-University Institute for Data Intensive Astronomy, Department of Physics and Astronomy, University of the Western Cape, Bellville 7535, South Africa; smacfarlane@uwc.ac.za

**Keywords:** mental health, awe, attention restoration theory, astronomy, nature-based interventions, African populations, feasibility pilot

## Abstract

**Highlights:**

**Public health relevance—How does this work relate to a public health issue?**

- Depression, anxiety, and stress-related disorders are rising globally, with South Africa facing a 91% mental health treatment gap that demands accessible, low-cost complementary interventions beyond conventional clinical services.
- Urbanization and light pollution are progressively eliminating access to dark skies, removing a natural restorative resource before its public health value has been fully understood or protected.

**Public health significance—Why is this work of significance to public health?**

- In the small, uncontrolled Glencairn cohort, lower group level depression, anxiety and stress scores were observed immediately after the retreat. Qualitative findings from both retreat settings identified experiences of calm, perspective taking and relational connection.
- Qualitative findings suggest that astronomy-based interventions may carry cultural resonance in African contexts, with participant accounts aligning with Ubuntu philosophy and indigenous knowledge systems.

**Public health implications—What are the key implications or messages for practitioners, policy makers and/or researchers in public health?**

- Program designers may benefit from prioritizing cognitive spaciousness, contextual onboarding, and relational anchoring when adapting astronomy-based wellbeing interventions for diverse populations and settings. However, dark-sky preservation and existing astronomical assets should be regarded as a potential future research and policy direction only, not yet as established mental health infrastructure.
- Should future controlled research support these early findings, dark-sky preservation and existing astronomical assets (e.g., SALT and SKA sites) may offer a foundation for low-cost, community-based wellbeing programs, a direction warranting further investigation rather than a demonstrated outcome of this pilot.

**Abstract:**

Depression, anxiety, and stress-related disorders continue to rise globally, with South Africa’s burden intensified by structural inequalities and a 91% mental health treatment gap, making accessible complementary interventions urgently needed. This exploratory mixed-methods pilot study examined the feasibility and acceptability of astronomy-based mental health support grounded in Attention Restoration Theory and awe research and sought a preliminary signal of change in psychological well-being. Two sequential retreat iterations combined guided naked-eye and telescope-based stargazing with nature immersion: a proof-of-concept peer camp (*n* = 19, Glencairn) and a family-focused retreat (*n* = 22 across nine family groups, Sutherland). The Depression Anxiety Stress Scale (DASS-21) was administered before (T1) and after (T2) the Glencairn retreat; qualitative data comprised the Most Significant Change focus groups (Glencairn) and asynchronous text-based interviews (Sutherland). Mean depression, anxiety and stress scores were lower at T2 than at T1. Because questionnaires were completed anonymously, individual T1 and T2 responses could not be linked. A valid paired analysis was therefore not possible, and inferential significance testing was not undertaken. These results represent descriptive cohort level patterns and should not be interpreted as evidence of within person change. Qualitative findings across both settings indicated calm, perspective shifts, and relational connection, while environmental novelty and family dynamics introduced competing cognitive demands at Sutherland. These preliminary findings suggest that astronomy-based interventions may support short-term psychological well-being and highlight three design considerations for implementation in African contexts, offered as provisional insights rather than established implementation principles with the design considerations being: cognitive spaciousness, contextual onboarding and relational anchoring.

## 1. Introduction

The intersection of astronomy and mental health draws upon two primary theoretical frameworks. Kaplan’s Attention Restoration Theory (ART) proposes that natural environments engage “soft fascination”, an effortless form of attention that allows depleted cognitive resources to recover [1,2]. The night sky offers a unique form of soft fascination in being engaging without demanding active attention, vast without being overwhelming. Researchers have extended ART’s application to astronomical environments, arguing that the vast, perspective-inducing qualities of the night sky engage restorative attention processes [3,4].

Complementing ART, research on awe provides a second framework. Keltner & Haidt [5] characterized awe as an emotional response to vast stimuli that challenge existing mental models. Subsequent studies demonstrate that awe reduces self-focus, quiets inner criticism, and increases prosocial behaviour [6,7]. The “Overview Effect” described by astronauts represents a prototypical awe experience that ground-based astronomy attempts to replicate [8]. The night sky is thus argued to elicit awe naturally by presenting humanity within an incomprehensibly vast universe [5].

### 1.1. Global Research Evidence

A substantial body of research supports nature exposure for mental health. Berman et al. [9] demonstrated that walks in natural environments improved cognitive performance compared to urban walks. Ohly [2] conducted a systematic review finding consistent evidence that natural environments facilitate recovery from directed attention fatigue. Bratman [10] provided comprehensive evidence that nature exposure produces measurable benefits for psychological health, including reductions in stress, anxiety, and depressive symptoms.

Recent developments specifically examining night sky engagement include the Night Sky Connectedness Index by Barnes [3], a 12-item scale measuring individuals’ connection to the night sky. Validation studies found that greater night sky connectedness was positively associated with mental health and happiness, independent of general nature connectedness. Notably, people in light-polluted areas felt less connected to the night sky, suggesting that access to dark skies is an important environmental determinant of wellbeing.

Direct astronomy-mental health studies remain limited but growing. Gankhuu et al. [11] found that engagement with the starry night sky helped mitigate stress and alienation among isolated nomads in Mongolia during COVID-19. Japanese research demonstrated that dark night sky images significantly reduced oxygenated haemoglobin in the right prefrontal cortex and increased psychological healing ratings [4]. Barragan and Meltzoff [12] linked the opportunity to view the starry night sky to human emotion and behavioural interest in astronomy.

### 1.2. The African Context: Implementation Without Evidence

The African continent faces substantial mental health challenges that make innovative interventions particularly valuable. Neuropsychiatric disorders rank third in their contribution to the overall burden of disease in Africa, with anxiety and mood disorders most prevalent among adults (8.1% and 4.9% respectively) and generalized anxiety at 11% for children and adolescents [13]. In South Africa specifically, nearly 40% of the population experiences symptoms of depression or anxiety, with a mental health service gap of 91% [13,14].

Despite these challenges, Africa hosts significant astronomy infrastructure, including the Southern African Large Telescope (SALT), and the MeerKAT radio telescope which will be integrated into the future Square Kilometre Array (SKA) [15]. The International Astronomical Union’s Office of Astronomy for Development (IAU-OAD), hosted in Cape Town, coordinates the most significant global effort linking astronomy to development; since 2012, the IAU has granted nearly EUR 1.5 million to over 200 projects across 112 countries [16]. The OAD’s Flagship Project 2: Astronomy for Mental Health explicitly aims to harness the inspirational potential of astronomy and use it as a tool for improving people’s mental health and wellbeing.

### 1.3. South African Research: Building the Evidence Base

South Africa stands at the forefront of astronomy-for-mental-health research on the continent. The Stellenbosch University CoCREATE Health Hub and IAU-OAD collaboration examine how structured stargazing sessions can support mental health, grounded in ART, Acceptance and Commitment Therapy principles, and awe research [16]. Early public communications from this programme of work have described participant reports of improved mood and a sense of “mental spaciousness” following guided stargazing [17]. The present article provides the first formal report of these pilot retreats.

Building on this collaboration between the OAD, Stellenbosch University (SU), Table Mountain National Park (TMNP), Guardians of the Deep NPC, and the Inter-university Institute for Data Intensive Astronomy (IDIA), the present study reports two pilot retreats: a proof-of-concept peer camp and an expanded family-focused retreat.

A critical insight from South African research is the importance of contextual adaptation. In settings where mental well-being is understood relationally (Ubuntu philosophy), interventions cannot rely on Western models of individualistic “escape.” The African concept of Ubuntu: “I am because we are”, reflects that individual well-being is inseparable from collective flourishing. Astronomy-based interventions therefore require deliberate, culturally grounded onboarding that addresses the socio-economic contrasts participants encounter.

### 1.4. The Research-Implementation Gap

Despite promising theoretical foundations and preliminary findings, significant evidence gaps remain. No systematic reviews specifically examining astronomy-based mental health interventions exist. No randomized controlled trials specifically testing astronomy interventions for mental health outcomes have been conducted in Africa. Existing studies are primarily pilot studies with small sample sizes, pre–post designs without control groups, or qualitative explorations. Optimal frequency, duration, and intensity remain unknown.

While NGOs, NPOs, and development organizations are actively implementing astronomy-for-development initiatives across Africa, academic research has not kept pace with practice. Most initiatives lack rigorous evaluation frameworks, and existing studies are concentrated in South Africa, leaving the rest of the continent unexamined. Limited evidence exists for specific populations including clinical populations, children and adolescents, elderly populations, refugees and trauma survivors, and across different cultural contexts.

### 1.5. Framing the Current Study

To advance beyond theoretical promise toward practical implementation, this study adopts an iterative pilot approach to explore the feasibility and potential effects of astronomy-based mental health interventions within the South African context. Recognising that effective interventions must account for both neurobiological mechanisms of restoration and relational understandings of well-being, the study examines how guided stargazing and nature immersion function across differing environmental and social contexts.

Two pilot iterations were conducted, varying key conditions including environmental setting and social configuration, to explore how these factors influence participant experience and perceived outcomes. This approach enables the identification of contextual and design considerations necessary for developing culturally responsive, community-oriented interventions in African settings.

This study contributes to the emerging field of environmental and nature-based mental health interventions by providing an early, exploratory mixed-methods examination of astronomy-based mental health support in an African context. Specifically, it aims to (1) assess the feasibility and acceptability of guided stargazing interventions, (2) generate preliminary evidence of short-term mental health outcomes, and (3) identify contextual and relational factors influencing participants’ experience of the intervention across differing environmental and social settings.

## 2. Materials and Methods

### 2.1. Research Design

This is an exploratory mixed-methods feasibility and acceptability pilot study, employing an iterative rather than a controlled-comparison design. Its primary objective was to establish the feasibility and acceptability of delivering astronomy-based mental health support within the African context. Due to its exploratory nature, no pre-specified feasibility indicators were established; instead, the feasibility of the intervention was assessed descriptively by considering the recruitment and retention of participants, whether the intervention could be implemented as intended and whether participants would engage with and tolerate it [18]. Secondary objectives were to obtain a preliminary signal of impact on psychological well-being (depression, anxiety, and stress) and to explore participants’ subjective and relational experiences, including family cohesion and connection. Consistent with the purpose of a pilot study, the quantitative outcomes are reported as preliminary indications to inform a future definitive trial rather than as confirmatory tests of efficacy.

The two retreats were conducted as sequential iterations of an evolving intervention rather than as a head-to-head comparison of pilots. The first retreat (Glencairn, Cape Town, South Africa) served as a proof-of-concept peer camp; insights from it informed the design and delivery of the second, family-focused retreat (Sutherland, Northern Cape, South Africa). Because the two pilots differ in place, participants, social context, and the measures available at each, they are presented as developmental stages in refining the intervention and its delivery, not as matched conditions to be statistically contrasted.

The study combined quantitative measures to assess changes in mental well-being with qualitative inquiry using the Most Significant Change (MSC) technique [19]. MSC was selected as it allows participants to articulate complex, subjective changes, such as shifts in perspective or feelings of awe, that predefined quantitative measures might miss. Family cohesion was assessed using asynchronous text-based interviews, collecting personal reflections on changes in family dynamics. The research team was involved in programme facilitation, and reflexive practices were used to critically consider how this may have shaped data collection and interpretation.

### 2.2. Setting and Site Selection

To ensure the interventions facilitated Attention Restoration Theory (ART) and awe, venues were purposely selected based on specific criteria outlined in the ethical protocol. First, locations required dark skies with minimal light pollution to ensure optimal naked-eye stargazing. Second, scenic and clutter-free natural settings were prioritized to enhance soft fascination and restorative potential. Third, venues required secure environments for nighttime activities and basic amenities to support group comfort. Finally, locations needed to be reachable for diverse participants while still providing a sense of being away.

1. Glencairn (Pilot A): A coastal mountain venue providing a green nature experience with moderate accessibility.
2. Sutherland (Pilot B): A remote, arid location hosting the Southern African Large Telescope (SALT), selected for its dark sky status and the contrast between the stark landscape and advanced scientific infrastructure.

### 2.3. Participants and Recruitment

Purposive sampling was used to recruit participants relevant to the study’s interdisciplinary focus. The total sample (*n* = 41) comprised two distinct cohorts corresponding to the intervention pilots. As this was an exploratory pilot, sample size calculations were not necessary; however, both the Glencairn (*n* = 19) and Sutherland (*n* = 22) cohorts’ sample sizes were consistent with recommendations for pilot and feasibility studies [20,21,22]. Participants were eligible if they voluntarily enrolled in one of the retreats and were able to provide informed consent. No formal exclusion criteria relating to mental health status were applied, as the intervention was designed as a community-based wellbeing program rather than a clinical intervention. Individuals who did not participate in the retreat activities or declined to participate in the research were excluded from data collection.

Glencairn Cohort: Participants were recruited from social, professional and academic networks, including field rangers from Table Mountain National Park and astronomy and psychology postgraduates from local universities. Institutions were sent invites to deliver to their staff/students and individuals contacted the research team to indicate their interest. Sutherland Cohort: Participants comprised nine family groups, who were recruited through Stellenbosch University’s internal advertising, providing a link to sign up for the retreat via Microsoft Forms. Eligibility criteria for the Glencairn camp included those over the age of 18 who were employed by Table Mountain National Park or students at the local universities. These institutions were chosen to provide some diversity of participants through already established networks. For the Sutherland retreat, participants were eligible if they were part of the Stellenbosch University network or their family members. These criteria were chosen due to ease of access to this population. Table 1 details the demographic characteristics of the participants.

### 2.4. Intervention Design

The intervention adopted an iterative developmental design. Rather than testing a static programme, the pilot tested different combinations of restorative variables. The core activity, which consisted of accessible astronomical observation using the naked eye and manual Dobsonian telescopes, remained constant. However, the environmental context and social composition were varied to understand how these factors influence the restorative process.

The Glencairn and Sutherland retreats shared two core elements: (a) guided astronomical observation and discussion, and (b) immersion in natural settings. These components were intentionally designed to align with ART principles: Being Away (psychological escape), Fascination (effortless engagement with nature’s “soft” stimuli), Extent (feeling immersed in a rich world), and Compatibility (alignment with personal goals). Table 2 outlines the specific combinations of variables tested across the two pilot settings.

#### 2.4.1. Glencairn

The Glencairn retreat was held over two days with an overnight stay at a local rotary camp. On arrival, participants completed the pre-survey and then were introduced to the retreat by four facilitators from the OAD and Guardians of the Deep, two local organisations that focus on the restorative effects of natural settings. Participants were then given the opportunity to explore the natural surroundings and engage with the other participants. Over the two days, participants were encouraged to connect with nature with guided meditations and nature walks, as well as connect with other participants with various games and during mealtimes. In the evening, participants engaged in a discussion about astronomy led by the facilitators, where they learnt more about the stars, the universe and specific constellations, particularly those which would be visible that night. This was followed by stargazing, both using the naked eye and Dobsonian telescopes (a portable telescope that stands independently using ground mount) to explore the night sky with guidance from the facilitators who provided information about what was visible. Following the night viewing, participants were encouraged to personally reflect on their experiences and how this has impacted them. The second day included guided meditation and the completion of the camp, where participants were split into groups to answer questions about their experiences, each led by one of the facilitators.

#### 2.4.2. Sutherland

The Sutherland retreat was held over a weekend, from Friday to Sunday. Participants arranged their own accommodation in and around Sutherland, and a central gathering point was established by the research team. On Friday evening, the group gathered at the central point for a fireside conversation led by the Director of the OAD and initial planning for the next day. Participants were introduced to some astronomical information and the background of the SALT. There was a portable telescope available for stargazing. The following day included a sunrise walk for those who were interested and then a short walk to Ou Lokasie, where participants learned more about the history of Sutherland. The afternoon was dedicated to visiting SALT; participants explored the visitor centre and then were taken up to the telescope plateau, where they were given a tour of the various telescopes and their history. This culminated in a sunset viewing and participants then returned to the visitor centre. The evening activities were held at the central meeting point, where families were given the chance to spend time together stargazing and using the telescopes. The retreat was then concluded with some reflections from the group.

### 2.5. Measures and Data Collection

Consistent with the iterative design described in Section 2.1, the assessment instruments were deliberately matched to the outcomes of interest in each iteration rather than held constant across the two settings. The first iteration (Glencairn) aimed to obtain a preliminary signal of individual psychological distress, for which DASS-21 provided an appropriate, locally validated standardized measure. The second iteration in Sutherland deliberately shifted the focus toward relational and family-cohesion outcomes. These are dimensions that an individual-level distress scale such as the DASS-21 is not designed to capture. For this reason, quantitative symptom assessment was not administered at that site, and qualitative methods better suited to exploring relational experience were used instead. A direct consequence of this design is that all quantitative inference in this study is confined to the Glencairn cohort. The DASS-21 results are not generalized to Sutherland, and the two pilots are not quantitatively compared.

Qualitative inquiry was used in both pilots, using the modality best suited to each (MSC focus groups at Glencairn; asynchronous text-based interviews at Sutherland). Table 3 outlines the distribution of instruments across the two pilots.

#### 2.5.1. Depression Anxiety Stress Scale (DASS-21)

DASS-21 [23] served as the primary measure to establish a quantitative baseline for general mental well-being during the Glencairn retreat. This 21-item instrument assesses current states of emotional distress across three subscales: Depression, Anxiety, and Stress. Items are rated on a 4-point Likert scale ranging from 0 (Did not apply to me at all) to 3 (Applied to me very much or most of the time). The DASS-21 was selected for its strong psychometric properties within the South African context [24], where it has demonstrated high internal consistency (Cronbach’s alpha 0.70 to 0.97). DASS-21 was administered on paper (and later captured digitally) and anonymously; no identifying codes linked to an individual’s pre- and post-intervention responses.

#### 2.5.2. Most Significant Change (MSC) Focus Groups

Qualitative data were collected from the Glencairn cohort using the MSC technique [19]. MSC is particularly suited to non-clinical interventions as it allows participants to define the value of the experience in their own terms. Three facilitated MSC focus groups, each comprising six to seven participants (total *n* = 19), were conducted in person on the final day of the retreat.

#### 2.5.3. Asynchronous Text-Based Interviews

Data from the Sutherland cohort was obtained through asynchronous text-based interviews conducted on WhatsApp (v2.25.23.73). Because participants attended as family groups and the second iteration focused on relational reflection, asynchronous text-based interviews were the most appropriate method, allowing family members to respond individually, at their own pace, and with greater privacy [25]. The five prompts were developed by the research team to elicit reflection on enjoyment, shared experience, family dynamics, and post-activity affect, informed by the study’s theoretical framework. The prompts were not formally validated (e.g., through Delphi procedures); this is acknowledged as a limitation.

### 2.6. Data Collection Procedure

Data collection at Glencairn occurred at two timepoints. At registration on arrival (T1), participants completed the demographic questionnaire and the DASS-21. On the final day (T2), participants repeated DASS-21, after which the three in-person MSC focus groups (Section 2.5.2) were conducted by the facilitators. At Sutherland, participants completed the demographic questionnaire at registration, and the five text-based prompts were distributed via WhatsApp over the weekend, with participants responding individually at their own pace.

#### 2.6.1. Focus Group

During the T2 focus group, participants were presented with three specific prompts designed to elicit narratives regarding restoration, nature, and astronomical engagement: Can you describe the most significant change you experienced during the retreat related to your mental well-being? How did the astronomy activities (e.g., stargazing, cosmic perspective discussions) affect your outlook on life or personal challenges? What, if anything, surprised you about your experience during the retreat?

#### 2.6.2. Text-Based Interviews

During the Sutherland retreat, the text-based interviews included five questions/prompts that were sent to participants over the course of the weekend.

- What has been your favorite part of the trip so far?
- Share your thoughts on yesterday when we were all together.
- What did you enjoy the most?
- What has the weekend done for your family?
- How are you feeling after the weekend’s activities?

### 2.7. Data Analysis

#### 2.7.1. Quantitative Analysis (Glencairn)

Participants completed the DASS-21 anonymously at two points: on intake (T1) and the final day of the retreat (T2). Because responses were anonymous, the item values at T1 and T2 could not be matched and so individual comparison between the two collection points was not possible. Therefore, findings are reported descriptively as differences in the cohort-level distributions of DASS-21 scores at T1 and T2 using measures of central tendency.

#### 2.7.2. Qualitative Analysis (Glencairn and Sutherland)

Participants engaged in focus group discussions on the final day of the Glencairn retreat; three separate groups were conducted, audio-recorded, and transcribed. Text-based interview questions received during the Sutherland retreat via WhatsApp over the course of the weekend were copied into a separate document to be analysed. There were 42 messages that were suitable for inclusion in the analysis process; some of these were from individuals and some on behalf of families with all nine families being represented.

The focus-group transcripts and text-based interview responses were analysed using reflexive thematic analysis, following the six phases described by Braun and Clarke [26]: familiarisation, initial coding, theme generation, theme review, theme definition and naming, and reporting. Coding was conducted using ATLAS.ti (v26.1.1) by one researcher (NT) with ongoing discussion to enhance interpretive depth with the entire research team. Analysis was primarily deductive, coding patterns relevant to the study’s theoretical framework (attention restoration, awe, cognitive load, and perspective-taking) while remaining open to inductive themes arising from the data. Consistent with a reflexive approach, inter-rater reliability statistics were not computed; instead, analytical rigour was pursued through engagement in reflexive practices described in Section 2.8. Several authors both facilitated the retreats and analysed the data; while this dual role affords interpretive closeness, it also risks confirmatory interpretation.

### 2.8. Sources of Bias and Reflexivity

Several potential sources of bias warrant acknowledgement. Self-selection bias is likely, as participants opted into a retreat they expected to enjoy. Self-reported outcomes are susceptible to social-desirability and expectancy effects, and the timing of the post-intervention assessment on the final day of the retreat may capture transient mood elevation rather than durable change. Another point to acknowledge is that multiple aspects were introduced in the retreats to elicit restorative effects, such as mindfulness, nature exposure, time away from responsibilities, and astronomy activities. This makes it difficult to isolate the contribution of every single component, which limits the ability to attribute any observed change to a single component.

Due to the exploratory nature of the study and the dual role played by many of the research team, reflexive practices were necessary and incorporated in the research process to acknowledge the researchers’ positionality and involvement in programme facilitation.

This was facilitated through debriefing sessions where the members of the research team reflected on their personal experiences of the retreats and how their backgrounds could have impacted data collection and the analysis. Additionally, during the process of data analysis, the initial themes generated were constantly reviewed and refined by members of the research team to ensure transparency.

## 3. Results

### 3.1. Feasibility Findings

As reported in Table 1, the total participant recruitment over both retreats was 41 participants, 19 at the Glencairn retreat and 22 at the Sutherland retreat. Out of those 41, all participants remained active in the research until completion. Engagement in the research process was high, especially in the Glencairn group where all participants completed the surveys and participated in the focus groups. In the Sutherland cohort, individual engagement was not so prominent, as the focus was more on family experiences. Because of this, some family groups would send a single response to the WhatsApp prompts, which was a summary from their entire family group. General engagement in the retreat activities was also promising, with all participants present for the astronomy activities at both retreats.

### 3.2. Quantitative Findings

Table 4 reports the descriptive DASS-21 subscale scores collected at T1 and T2 for the Glencairn cohort (*n* = 19 at each time point). The average scores (mean and median) were lower at T2 than T1 on all three subscales, showing differences in the two group distributions. These results suggest that reported group levels of anxiety, depression and stress were generally lower following the retreat than at intake. The largest observed mean group difference was on the stress subscale (−3.42), followed by anxiety (−3.21), then depression (−2.37). Standard deviations were relatively large in relation to the mean scores, particularly for anxiety and depression, suggesting considerable variability in the reported scores.

### 3.3. Qualitative Findings—MSC Focus Groups (Glencairn)

Three focus groups were conducted with the participants at the end of the Glencairn retreat. The discussions explored the participants’ perceptions and experiences of the programme and how this impacted their mental wellbeing. The themes that arose from these conversations are presented below.

#### 3.3.1. Glencairn Themes

##### Sense of Calm and Stress Release

Participants described the retreat environment as creating a calming atmosphere where they could let go of stress and gain respite from the demands of day-to-day life. One participant shared, “*I was actually at peace*”, whereas another mentioned,

“*It just kind of slows you down a bit and you get back into your normal rhythm. Instead of being like this, like you’re in a hamster wheel, just going on to the next thing, not dealing with what you actually need to deal with, just keep running away from it*”.

These accounts suggest that the retreat environment facilitated a process of slowing down the mind and body, allowing for a more balanced approach to daily activities and, consequently, a reduced mental load.

##### Enhanced Sense of Community and Connection

Some participants highlighted their experience of building connections with others and feeling accepted in the group as something that challenged their personal ideas of themselves. One shared,

“*This is the first time that I’ve, like, I’ve had such a positive experience with you guys, and it’s really helped me, sort of, shift that perception of myself was so negative*”.

Another participant highlighted the value that can emerge from coming together as a group, “*I realised that we gain more experience of interacting with each other, of learning the new things from each other. And its new experience now that we learn about many things from different aspects that they know*”. This shows that some participants experienced profound feelings of connection with other people during the retreat and this impacted them positively.

##### Expanded Perspective

Several participants explained how they felt they had gained a greater perspective on their lives and on personal issues they are dealing with. One shared, “*there’s, like, a sense of, like, we’re all just tiny little animals on this rock. And that universal truth and the universality of it and the spirituality of it definitely hit home for me when we’re all standing there*”.

Another participant explained,

“*The more you’re zooming into whatever you’re looking at, you keep getting smaller and smaller. Your problems keep getting smaller and smaller*”.

This shows the impact that being faced with the enormity of the stars and the galaxy had on the way individuals viewed their own lives and problems. Through understanding the scale of the universe, there was a sense that by focusing so intensely on specific aspects of life, one loses perspective about what is important.

##### Cultural Significance

Another theme that was presented was the way the stars align with culture, and that people connected to their cultural knowledge to glean personal significance from the experience of looking at the stars.

One individual explained, “*That they’re not just stars. There’s a message within each star. Each star has its own story and all of that. And that can actually be applied in our own lives*”.

Another participant shared, “*We thought the stars are just the stars. And we call it… There’s a star that we say… I don’t know what is the English name. It’s coming from the east. When the sun is rising, it stands up all the way to this guy’s nose. And there’s a star that comes from that star. Okay. When it’s about to sunrise, then we call it… Kwezi. That means it’s a very bright star*”.

These accounts highlight the different ways in which people make sense of the stars and how to each person it means something different. It shows the value of cultural meaning and how it could transform the night sky into a socially resonant space. The importance of the stars in South African cultures implies that the sky can represent something deeper and allow a personal connection to something greater.

##### Personal Understanding

Another aspect that came across was the way that participants had gained a greater sense of personal understanding from their time at the retreat. One participant shared, “*What surprised me about this whole experience is how high strung I am. It doesn’t come across that way, but in the background, there is chaos*”. This shows that the process of slowing down during the retreat had appeared to increase participants’ awareness of their usual levels of tension and cognitive activity. Another said, “*…at first I was thinking, wow, this is exactly the stuff that I’ve been thinking about. My position, doing astronomy, what does that mean? Why am I doing it? Do I really love what I’m doing? All that kind of stuff*”. This indicates that the retreat helped to facilitate these questions for the participant, leading them to try to understand more deeply their reasons for making certain decisions in their life and providing the mental space to be able to truly reflect on these decisions.

### 3.4. Qualitative Findings—Text-Based Interviews (Sutherland)

Over the course of the weekend retreat, five questions were asked using WhatsApp Messenger and individuals were asked to respond in their own time. The general themes identified are presented below.

#### 3.4.1. Sutherland Themes

##### Ideas About Family

One theme that came across was how participants thought about their concept of family, one shared, “*when I left for weekend, I didn’t conceptualise the family beyond the 2 of us. Realised the family was larger and included family and friends—from 2 to 8 and a half with toddler. Recognition of importance of families*”. This account reflects how taking time together as a family unit appeared to highlight the importance of family and how family can mean more than the traditional sense. Another participant wrote that, “*The weekend gave the family a chance to share time, stories and each one got to have time together and individually to explore.*” This participant described how the retreat appeared to create opportunities for families to spend quality time.

##### Importance of History

Participants reflected on the continued influence of South Africa’s historical context within the Sutherland setting. One of the participants wrote, “*The sense of race and class is still so divided*”, reflecting on the ongoing divisions related to race and class. Another individual replied, “*our word is ‘development’ the good, the bad, the ugly, the untold and mis-told truths and how interesting the dynamic is in an environment like South Africa*”. These accounts suggest that participants situated their experience within the broader historical and structural realities that are still relevant today in South Africa.

##### Logistics

A prominent theme revolved around the logistical issues that had been experienced by participants. One of the participants had reflected that, “*I think it would be helpful to build in time for reflection, which we can also do as a family after each activity or at the end of each day*”. Another individual shared that they felt, “*More prompts at getting to know each other would have made it feel less isolating*”. One participant suggested that the schedule for the weekend was too busy, “*Centre session was a bit long/could have had better time management—program bit too full. Negative impact on the rest of the time as having to communicate changes*”. These aspects were important to note as areas for improvement. The participant feedback highlighted that planning multiple activities over a short period was counterproductive and added to stress and fatigue. Also, it was noted that when bringing together multiple groups of people, there needs to be more effort made to integrate them and encourage people to get to know each other.

##### Mental Restoration

One concept that was communicated was the experiences of mental restoration that the participants had experienced. One shared, “*The SALT tour and stargazing was for me a good time to appreciate having an opportunity to see what we saw and to reflect on how busy I let life get with many things*”. This was iterated by another participant who said, “*I wanna take back the calmness Sutherland has*”. Showing that the activities and the environment had prompted a sense of calm and reflection. Another individual wrote, “*A sense of appreciation being here just being part of something bigger*”, which reiterated that the weekend had encouraged perspective building.

##### Environment Appreciation

A clear theme that arose was the appreciation that participants had shown for the natural environment. One participant shared “*Privilege to be part of Milky Way at this time of year when the core is visible at night in southern hemisphere*”. Another individual wrote, “*Awed by the beauty and the isolation. And the determination of life to survive.*” This highlighted the way that participants connected with the environment and that it allowed for deeper self-reflection and contemplation.

## 4. Discussion

The findings from this exploratory pilot study provide preliminary descriptive findings regarding the feasibility and potential restorative effects of astronomy-based interventions, while highlighting important contextual and design considerations within the South African setting.

In the Glencairn cohort, the familiar coastal-natural setting, peer social container, and guided astronomical observation were associated with short-term restorative changes. Descriptive analysis of DASS-21 scores showed lower mean and median values at T2 compared to T1 across all subscales. These findings are consistent with Attention Restoration Theory [1] as a descriptive pattern. The theory describes how being surrounded by a natural environment such as the night sky can provide respite from attentional fatigue through fascination or involuntary attention. This helps to restore depleted mental focus without requiring any extra effort from the brain [2]. Qualitative accounts further complement this picture: participants described mental calm, release from the “hamster wheel” of daily life and expanded perspective (“our problems keep getting smaller”), which align conceptually with ART’s core elements of Being Away, Extent, and Compatibility.

**Figure 1:**
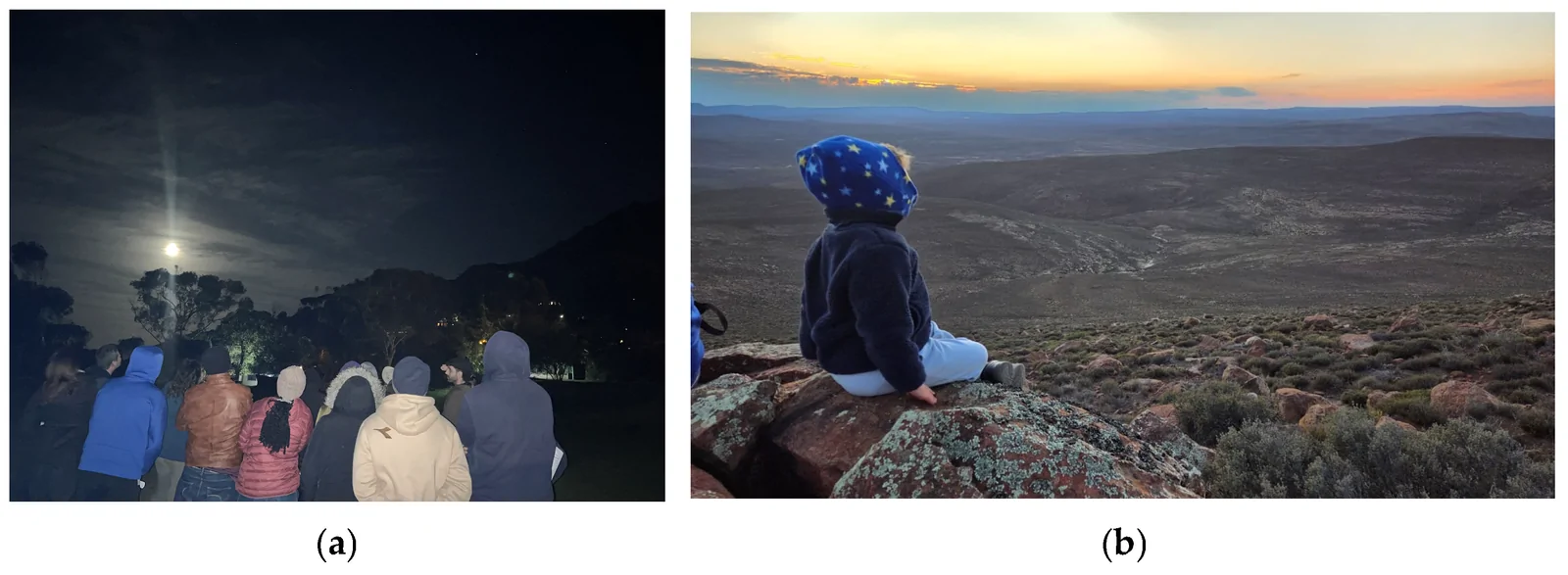
(**a**) Group stargazing at the Glencairn retreat; (**b**) view from the Southern African Large Telescope (SALT) in Sutherland.

Awe may operate as a complementary mechanism. The cosmic scale elicited perspective-taking that appeared to diminish self-focus and rumination (according to qualitative participants’ accounts); this is consistent with established awe literature and the Overview Effect analogue [8]. As illustrated in Figure 1, participants encountered the night sky through group stargazing during the Glencairn pilot (a) and through engagement with the astronomical environment surrounding the Southern African Large Telescope (b) during the Sutherland pilot. Cultural resonance themes further distinguished the experience: participants spontaneously linked stellar observation to indigenous knowledge (e.g., “Kwezi”), bridging scientific wonder with personal and collective meaning-making. This cultural integration suggests the night sky could function not merely as a restorative stimulus but as a culturally resonant space in African contexts.

By contrast, the Sutherland retreat showed how adding more layers of complexity can diminish these benefits. While the astronomical highlights, such as the SALT tour and stargazing, still elicited awe, the other elements introduced competing cognitive demands. Family systems, long-distance travel, the novelty of the dry landscape, and sharp socio-economic contrasts all played a role. Logistical fatigue, a densely scheduled program, and reflection on historical divisions drew attention away from restoration and toward active processing of demanding realities.

These findings are aligned with the African philosophy of Ubuntu, which is grounded in the idea of “I am because we are”. This implies that individual wellbeing is dependent on the wellbeing of the group. In this light, shared nights under the stars can strengthen family bonds, build a feeling of communal safety, and create moments of togetherness that participants experienced as restorative. At the same time, such group experiences require skilled facilitation so that family dynamics or differing viewpoints do not become additional sources of stress.

The different qualitative patterns observed across the two developmental iterations extend this theoretical framework by suggesting that the night sky’s restorative potential is not automatic; during this research, the programme design appeared to be a key aspect in protecting cognitive spaciousness and the attentional capacity that allows restoration to occur. However, the two settings used different measures and were not designed as comparable conditions, so direct comparison of outcomes is not appropriate. Excessive novelty, dense scheduling, or unfacilitated engagement with social and political realities show how awe can shift from being restorative to demanding, particularly in settings marked by inequality.

The qualitative findings, combined with the descriptive cohort-level DASS-21 pattern from Glencairn and other feasibility data, suggest a provisional account of how astronomy-based retreats may be feasible and acceptable to support mental restoration in South Africa. The interaction between restorative and load-inducing factors is offered as a provisional hypothesis for testing rather than as a mechanism established by these data, and forms the basis for the three considerations set out in Section 5.

The study, therefore, offers practical information to guide astronomy-for-development programs. Importantly, these interventions need not rely exclusively on major observatories; instead, community astronomy initiatives, mobile telescopes and existing outreach programs may provide scalable pathways for implementation should future controlled research support these preliminary descriptive findings. It highlights those culturally sensitive adjustments, such as building strong relational anchors and easing people gently into the experience, may be important when moving beyond the more individualistic Western retreat model.

Notwithstanding its preliminary nature, this study contributes a set of provisional intervention design considerations that may extend beyond the specific context: cognitive spaciousness, contextual onboarding, and relational anchoring offer potential directions for developing culturally responsive, community-oriented mental health interventions that integrate environmental and social dimensions of well-being. Further controlled investigation is required to establish these as actionable principles.

This study has several limitations. First, the absence of a control group precludes causal inference; observed changes may reflect general retreat effects such as removal from daily stressors, social connection, and environmental change rather than astronomy-specific mechanisms. Second, the samples were small and purposively recruited, restricting generalizability, and because the DASS-21 was administered only in the Glencairn cohort, all quantitative findings are confined to that setting and are not generalized across the two retreats. Third, DASS-21 responses were collected anonymously and could not be linked across time points; analyses therefore compared differences in group-level distributions rather than matched pairs, which precludes conclusions about individual change and means that differences in who responded at each time point cannot be excluded as a contributing factor to the observed differences. Fourth, outcomes were assessed immediately post-intervention only, so the durability of any benefit is unknown. Fifth, self-reported outcomes are vulnerable to social-desirability and expectancy effects, and the five text-interview prompts were not formally validated. Lastly, the research team’s involvement in program facilitation may have influenced data collection and interpretation. Even so, the iterative mixed-methods approach, the real-world African settings, and the correspondence between the Glencairn quantitative pattern and the qualitative accounts from both settings provide a credible starting point for more controlled investigation. The study design does not isolate the contribution of astronomical observation from these other contextual factors.

## 5. Conclusions

This exploratory pilot study provides preliminary descriptive findings regarding the potential contribution of astronomy-based interventions to short-term psychological well-being in South Africa. Across two distinct retreat contexts, the findings suggest that accessible dark-sky experiences could contribute to attention restoration and promote perspective shifts and were accompanied by lower reported short-term distress in the one cohort where distress was measured, at least under supportive conditions.

Qualitative findings imply that the value of awe may be context-specific and that its potential could be dependent upon program design, as well as the facilitation of a contained and protected space. Three ideas arose as potential considerations for future research: (1) cognitive spaciousness, meaning adequate unscheduled time for reflection and integration; (2) contextual onboarding, meaning transparent preparation for the environmental, social and socio-economic realities participants will encounter; and (3) relational anchoring, meaning skilled facilitation so that group dynamics become a source of communal safety rather than stress. These are offered as directions for future research rather than as established design principles.

Should future controlled research support these preliminary descriptive findings, preserving access to dark skies may merit consideration as a potential component of public health strategy. This may be particularly relevant in African contexts, where mental health services remain under-resourced and astronomical heritage is both extensive and culturally significant.

### Way Forward

Future research should employ larger and diverse samples, longitudinal designs and controlled comparisons (including clinical, youth, and trauma-affected groups), and validated tools such as the Night Sky Connectedness Index [3]. Dose–response studies and cost-effectiveness analyses will clarify optimal parameters. Practically, astronomical bodies and partners could explore facilitator training, open-source toolkits, and should the evidence base strengthen, advocacy that considers dark-sky preservation as a potential mental-health resource. While these findings suggest preliminary descriptive patterns, further rigorous controlled evaluation is required before considering implementation at scale or informing large-scale policy.

In resource-constrained settings where access to formal mental health services is limited, integrating environmental and culturally resonant approaches into community health ecosystems represents a promising direction for public health innovation.

## Figures and Tables

**Table 1:** Participant demographic characteristics.

Measure	Glencairn (*n* = 19)	Sutherland (*n* = 22)	Total (*n* = 41)
Age (Years)			
18–25	1 (5.26%)	1 (4.55%)	2 (4.88%)
26–35	10 (52.63%)	1 (4.55%)	11 (26.83%)
36–45	6 (31.58%)	12 (54.55%)	18 (43.90%)
46–55	2 (10.53%)	3 (13.64%)	5 (12.20%)
56+	0 (0%)	5 (22.73%)	5 (12.20%)
Gender			
Female	10 (52.63%)	13 (59.09%)	23 (56.10%)
Male	9 (47.37%)	9 (40.91%)	18 (43.90%)

**Table 2:** Intervention logic and combinations tested.

Variable	Pilot A: Glencairn	Pilot B: Sutherland
Environmental Setting	Green/Coastal: Lush, familiar nature designed to support restoration via soft fascination (ART).	Arid/Stark: Remote, high-contrast landscape offering deep novelty and a profound sense of being away to stimulate reflection.
Astronomical Context	Direct Engagement: Stargazing focused purely on the visual experience using naked eye and manual Dobsonian telescopes.	Contextual Contrast: Accessible stargazing (Dobsonian) juxtaposed with a daytime excursion to the Southern African Large Telescope (SALT), grounding the personal viewing experience within the context of Big Science.
Social Container	Individual/Peer: Experience focused on individual self-reflection within a loose professional network.	Family Systems: Experience focused on family connection, testing the impact of family cohesion on the restorative process.

**Table 3:** Summary of measures administered by pilot setting.

Measure	Domain Assessed	Pilot A: Glencairn (Individual/Peer)	Pilot B: Sutherland (Family/High-Tech)
Demographic Questionnaire	Age, gender	Yes	Yes
DASS-21	Depression, anxiety, stress (distress)	Yes	No
MSC Focus Groups	Subjective change and perspective	Yes	No
Asynchronous text-based interview	Personal reflections on family dynamics	No	Yes

Note: instruments were tailored to each iteration’s outcomes of interest. The DASS-21 was administered only at Glencairn, so quantitative findings are confined to that cohort and the two pilots are not quantitatively compared.

**Table 4:** Descriptive DASS 21 subscale scores before and after the Glencairn retreat.

Subscale	T1 Mean (SD)	T1 Median (IQR)	T2 Mean (SD)	T2 Median (IQR)	Observed Difference (T2 − T1)
Anxiety	5.84 (3.29)	6.00 (6.00)	2.63 (3.00)	1.00 (5.00)	−3.21
Depression	4.84 (3.13)	5.00 (5.00)	2.47 (2.84)	1.00 (4.00)	−2.37
Stress	8.05 (3.95)	8.00 (4.00)	4.63 (4.39)	3.00 (6.00)	−3.42

Note: The same cohort (*n* = 19) completed the DASS-21 at both T1 (before the retreat) and T2 (immediately after the retreat). Because questionnaires were completed anonymously, individual T1 and T2 responses could not be linked; the values above therefore describe two cohort-level distributions rather than matched pairs. No inferential testing was undertaken, and these figures do not provide evidence of individual-level change. (SD = standard deviation; IQR = interquartile range).

## Data Availability

The raw data supporting the conclusions of this article will be made available by the corresponding author on request.

## Abbreviations

The following abbreviations are used in this manuscript:

ART	Attention Restoration Theory
DASS-21	The Depression, Anxiety and Stress Scale—21 Items
MSC	Most Significant Change
IAU-OAD	International Astronomical Union’s Office of Astronomy for Development
SALT	The Southern African Large Telescope
MeerKAT	The South African MeerKAT radio telescope. A precursor to the Square Kilometer Array (SKA) telescope
SKA	Square Kilometre Array
TMNP	Table Mountain National Park
IDIA	Inter-university Institute for Data Intensive Astronomy
CoCREATE	Creative Research for Equity and Transdisciplinary Knowledge Exchange
